# Investigational New Drug-enabling studies to use genetically modified mesenchymal stromal cells in patients with critical limb ischemia

**DOI:** 10.1093/stcltm/szae094

**Published:** 2025-02-26

**Authors:** Amin Cressman, Bryan Le, David Morales, Won-Shin Yen, Fang-Ju Wu, Nicholas H Perotti, Brian Fury, Jan A Nolta, Fernando A Fierro

**Affiliations:** Stem Cell Program, University of California at Davis, Sacramento, CA 95817, United States; Stem Cell Program, University of California at Davis, Sacramento, CA 95817, United States; Stem Cell Program, University of California at Davis, Sacramento, CA 95817, United States; Taiwan Bio Therapeutics, 5F., No. 66, Shengyi 2nd Rd., Zhubei City, Hsinchu County, Taiwan; Taiwan Bio Therapeutics, 5F., No. 66, Shengyi 2nd Rd., Zhubei City, Hsinchu County, Taiwan; GMP Facility, University of California at Davis, Sacramento, CA 95817, United States; GMP Facility, University of California at Davis, Sacramento, CA 95817, United States; Stem Cell Program, University of California at Davis, Sacramento, CA 95817, United States; Stem Cell Program, University of California at Davis, Sacramento, CA 95817, United States; Department of Cell Biology and Human Anatomy, University of California at Davis, Sacramento, CA 95817, United States

**Keywords:** mesenchymal stem cells (MSCs), angiogenesis, bone marrow stromal cells (BMSCs), cellular therapy, clinical translation, gene therapy

## Abstract

Mesenchymal stromal cells (MSCs) have been tested in multiple clinical trials to treat peripheral artery disease, especially the more severe form called critical limb ischemia. However, MSCs have often not met the expected efficacy endpoints. We developed a more potent therapeutic by genetically modifying MSCs to overexpress Vascular Endothelial Growth Factor (VEGF-A165). Here, we report preclinical studies submitted to the Food and Drug Administration (FDA) as part of our Investigational New Drug submission package. In vitro studies included the characterization of cell banks, transcriptome and secretome analysis, and in vitro potency assays. In vivo studies using immune-deficient NSG mice include dose-finding efficacy studies using a Matrigel plug model, cell retention studies, measurements of circulating VEGF, and toxicology studies to rule out severe adverse events. Our results suggest both the safety and efficacy of MSC/VEGF and support a first-in-human clinical trial to test this new combined cell/gene therapy.

## Graphical Abstract

**Graphical Abstract:**
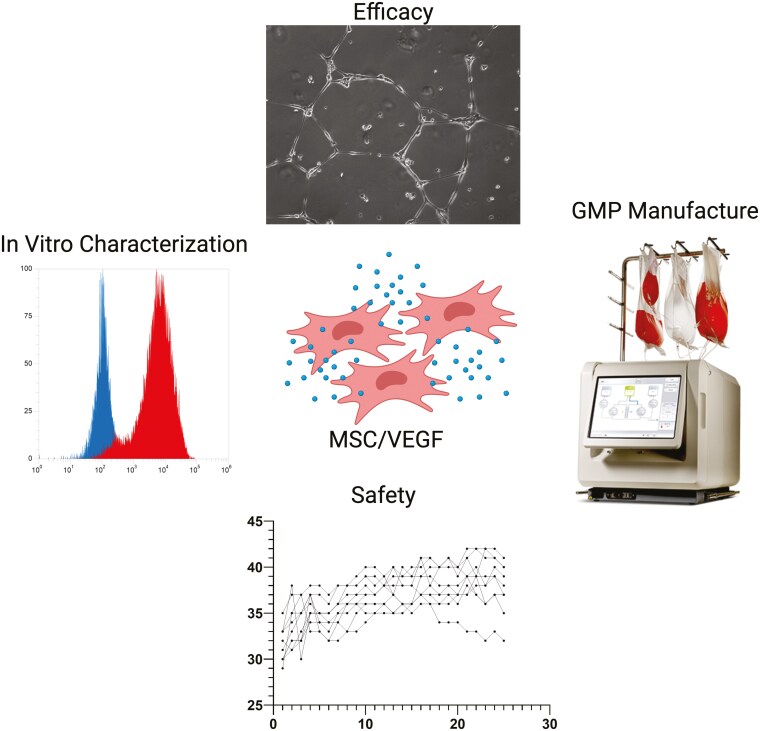

**Significance Statement**

Here, we describe our preclinical studies in preparation for an Investigational New Drug submission, to test mesenchymal stromal cells overexpressing VEGF for the treatment of critical limb ischemia.

## Introduction

Critical limb ischemia (CLI) is the most advanced phase of lower extremity peripheral artery disease (PAD), which leads to severe blood flow obstruction to the lower limb. This results in chronic pain that can advance to tissue necrosis, ulceration, or gangrene and carries a high risk of limb amputation, cardiovascular events, and even death. An estimated 6.5 million people in the USA, Europe, and Japan are affected by CLI,^1^ and the risk of lower limb amputation especially in nontreatable patients is notably high (10%-40%).^2^ Around one-third of CLI patients are not suitable for surgical or endovascular revascularization and the current limb-saving treatments have not reduced amputation rates as expected. Improved strategies focused on microvascular regeneration, such as targeted angiogenesis to restore blood flow to ischemic tissues, are therefore needed.^3^

Several clinical trials have used mesenchymal stromal cells (MSCs) based on their proangiogenic effect for the treatment of CLI^4-6^ and there is a substantial amount of evidence on the safety of MSC administration in humans.^7-10^ However, unmodified MSCs have often not met the expected therapeutic efficacy endpoints, especially for the most severe cases of CLI. Therefore, we envisioned genetically modifying MSCs to enhance their angiogenic potential by expressing supraphysiological levels of the key angiogenic factor VEGF. The intent, however, is to maintain the excellent safety profile of MSCs, by minimizing lentiviral insertions and thoroughly excluding autocrine effects due to transgene expression.

We have previously reported proof-of-concept data in animals supporting the superiority of MSCs overexpressing VEGF (MSC/VEGF) over unmodified MSCs to promote angiogenesis.^11-13^ However, thorough preclinical safety and efficacy studies remained necessary prior to initiation of a clinical trial. An Investigational New Drug (IND) package comprises 3 main sections: (1) Chemicals, Manufacturing, and Control (CMC), which includes a detailed description of product manufacturing, sterility tests, and all other required certification, (2) Clinical Protocol, describing in detail the intended clinical trial, and (3) Preclinical Pharmacology and Toxicology studies. Here, we report the results of the latter, with only a brief description of the manufacturing process and the intended clinical use. Our results suggest that MSC/VEGF are robustly angiogenic and that in the adequate dose, it will be as safe as unmodified MSCs.

## Methods

### Generation of MSC/VEGF cell banks

All manufacturing methods were performed under Good Manufacturing Practices (GMP) conditions, following standard operating procedures and using GMP-compliant (cGMP) reagents. For the bone marrow aspirate, a suitable donor was identified at UC Davis following our IRB protocol. The volunteer donor was healthy and tested for HIV-1 and 2, HTLV 1 and 2, Hepatitis B and C, treponema pallidum, CMV, and in addition, West Nile virus (due to the use of regional donors from Northern California). MSCs were isolated from the bone marrow aspirate following the standard technique based on plastic adherence. In brief, whole bone marrow aspirates were seeded into tissue culture flasks and cultured with regular medium changes (MEMalpha supplemented with 10% fetal bovine serum; FBS) every 3-4 days. As for most IND submissions,^14^ we chose to use qualified FBS for the expansion of MSCs. Once cells reached the desired confluency, cells were lifted using TrypLE and replated into for 2 additional passages. At passage 2, cells were cryopreserved at 5 million cells per mL in CS10 freezing medium (CryoStor) using a controlled rate freezer.

To manufacture the VEGF lentivirus, Lenti-X HEK-293T cells were transfected using the 4-plasmid system: VEGF,^12^ and the LentiHelper plasmids GAG-POL, REV, and VSV-G (all manufactured at cGMP grade by Genescript). To determine functional viral titer, MSCs were transduced with increasing volumes of lentivirus and tested for both vector copy number (VCN; determined by RT-PCR) and VEGF secretion (measured by ELISA). Based on the ELISA results, which were conducted as a safety measure to ensure VEGF secretion levels remain below 100 pg/mL/24 h/1000 cells,^15^ it was determined that 25 000 cells should be transduced with 0.04 µL of the specified lentivirus. The exact viral insertion sites were not characterized, since these are expected to vary from cell to cell. However, previous work demonstrated the integration stability (lack of genetic rearrangement) using this lentivirus.^12^

**Figure 1.**
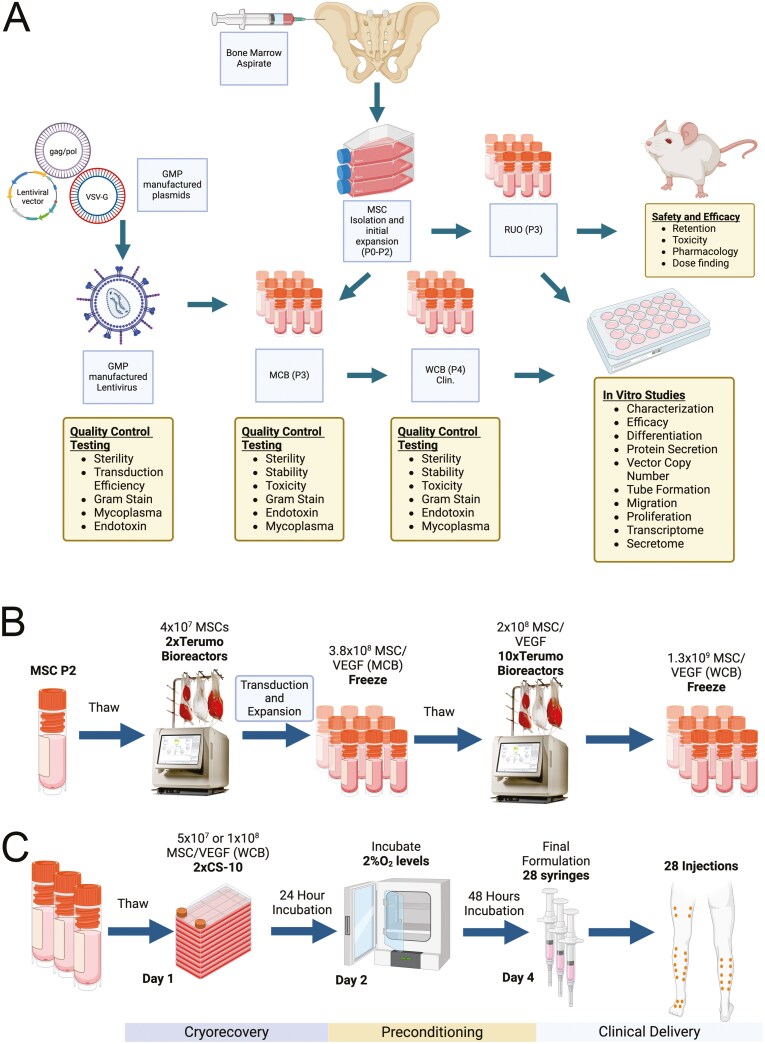
Schematic overview of manufacture of MSC/VEGF. (A) Steps for the manufacture of MCB and WCB of MSC/VEGF, highlighting quality control tests and experiments. (B) Key steps in the transduction and expansion of MSCs to generate the number of cells indicated of the final Clin-MSC/VEGF product. (C) Preconditioning and final formulation of MSC/VEGF for intended clinical use.

For the next steps, MSCs were manufactured using the Quantum Cell Expansion System (Terumo BCT, Inc) (Figure 1A and B), which is a bioreactor that uses a perfusion continuous flow media system. To prepare for bioreactor setup, first the bioreactor hollow fibers were coated with fibronectin (5 mg dissolved in 200-mL PBS). Then, MSCs were seeded at 2 × 10^7^ cells/bioreactor and cultured using MEMalpha + 10%FBS, at 37 °C, and 5% CO_2_. Approximately 16-24 hours after seeding the MSCs into the Quantum devices, transduction of the cells was performed, by adding high-titer VEGF lentiviral vector and protamine sulfate (20 μg/mL). The next day, the virus-containing medium was removed from the cartridges and cells were allowed to grow for additional 4 days. During this expansion, new media was continuously perfused through the cartridge at an initial flow rate of 0.2 mL/min, adjusted based on lactate and glucose concentrations to maintain physiological conditions supporting optimal cell growth until harvest (the cartridge contains 200-mL total volume). At harvest, cells were detached using TrypLE and cryopreserved using CS10 freezing medium at 5 × 10^6^ cells/mL in Crystal Zenith (CZ) vials (4 mL each, 20 million cells). At each round, approximately 2 × 10^8^ cells were harvested from each bioreactor. These cells (MSC/VEGF) were either an intermediate product for the generation of research use only (Ruo-MSC/VEGF) or became the master cell bank (MCB) for the Clinical Use (Clin) MSC/VEGF lot.

To further expand MSC/VEGF to generate Ruo-MSC/VEGF and the working cell bank (WCB) or Clin-MSC/VEGF, cryopreserved vials of MSC/VEGF (Ruo- or MCB) were retrieved and processed as for the MCB expansion process. For the generation of the WCB, 10 vials of MCB (containing 20 million cells each) were loaded into 1 bioreactor per vial, using 10 Quantum devices in total. All 10 Quantum Hollow Fiber Bioreactors were run using the identical expansion program settings on each device. The same culture media and downstream adjustments were replicated across all devices in the same way as the process that was used to generate the MCB. At harvest, cells from all 10 bioreactors were released, combined, washed, counted, and resuspended at 5 × 10^6^ cells/mL in CS10 freezing medium. Cells were divided into 5-mL aliquots (2.5 × 10^7^ cells per vial), cryopreserved using a controlled rate freezer, and stored in vapor phase of liquid nitrogen. For all cell banks generated in Quantum devices, glucose and lactate levels were monitored daily (Supplementary Figure S1). Ruo-MSC/VEGF were generated in the same way but using only 2 Quantum devices.

In our proposed clinical use, the final drug product (DP) is made from WCB cells that undergo hypoxic preconditioning (Figure 1C). Here, WCB vials are thawed 4 days before final DP release and seeded into multilayered CellSTACK tissue culture flasks using 1 vial (2.5 × 10^7^ cells) per flask. For low-dose treatments, 2 cryovials will be thawed and plated; for high dose, 4 cryovials. Cells were cultured in normoxic conditions for 18-20 hours, received a complete medium change, and then further cultured in hypoxic conditions (37 °C, 5% CO_2_, 2% O_2_) for an additional 48 hours. On infusion day, cells are lifted with TrypLE, concentrated, washed with DPBS, and resuspended in saline solution with 5% human serum albumin (HSA) at a concentration of 3.57 × 10^6^ cells/mL in a total volume of 14 mL (5.0 × 10^7^ cells in 28 syringes) for low dose or 7.14 × 10^6^ cells/mL (1.0 × 10^8^ cells in 28 syringes) for high dose. Cells are loaded into syringes at 0.5 mL per syringe. For all in vivo studies described here, cells were preconditioned in hypoxia using the same time intervals. The vehicle for injection into mice was saline solution +5% HSA.

### Determination of VCN per cell

The number of vector copies was determined in the RUO, MCB, and WCB by real-time PCR and dPCR utilizing Taqman Probes. Total genomic DNA was first extracted using a Quick-DNA Miniprep Plus Kit (Zymo) and PCR was performed measuring human Albumin (hAlb) (ALB forward: 5'-GCTGTCATCTCTTGTGGGCTGT-3' ALB reverse: 5'-ACTCATGGGAGCTGCTGGTTC-3' ALB probe: 5' HEX CCTGTCATG/ZEN/CCCACACAAATCT-CTCC-3IABkFQ-3' to detect total number of cells). We utilized primer/probe targeting WPRE (WPRE forward: 5'-CCGTTGTCAGGCAACGTG 3' WPRE reverse: 5'-AGCTGACAGGTGGTGGCAAT 3' WPRE probe: 5'-FAM-TGCTGACGCAACCCCCACTGGT-MGB-NFQ-3') to detect viral insertions. In real-time PCR method, using a standard curves of plasmid DNA containing the respective sequences, a total number of cells and an average number of viral copies per cell were quantified.

### Detection of secreted VEGF levels

To measure VEGF in supernatants, 5 × 10^5^ cells (all conditions in passage 5) were plated in 75 cm^2^ tissue culture flasks. The next day media was changed to 7 mL of MEMalpha + 10% FBS and subsequently harvested 24 hours after. VEGF levels were measured using a Human VEGF ELISA Kit (Biolegend), following manufacturer’s instructions. To measure VEGF in mouse blood, blood was collected from euthanized mice from the thoracic cavity and stored at −80 °C. VEGF was measured using a commercial ELISA kit (R&D Systems). To test the sensitivity and specificity of this kit, murine blood from untreated mice was spiked with recombinant human VEGF.

### Measurements of surface markers

Flow cytometry was used to measure surface markers in MSCs. Here, cells in suspension were incubated for 30 minutes at 4 °C with antibodies (all diluted 1:100 in PBS, all from BD Pharmingen). Cells were then washed once with PBS and immediately measured by flow cytometry using Attune NxT (Life Technologies). Positive or negative signals were determined by overlap with a respective negative control (unstained cells).

### Proliferation assays

To determine proliferative potential of the MSC/VEGF product compared with unaltered MSCs, cells were seeded into 12-well plates at 1000 cells per cm^2^ in triplicate. Every 3 days, cells were lifted using trypsin and counted using Trypan Blue exclusion dye and hemocytometer. Cells received medium changes every 2 days.

### Differentiation assays

To assess osteogenic differentiation of the clinical product compared with untransduced MSCs, MSC/VEGF cells were plated at 10 000 cells per cm^2^ in triplicate into 12-well plates and cultured with osteogenic medium, receiving medium changes every 3-4 days. Osteogenic medium contains standard culture medium supplemented with 0.2-mM ascorbic acid, 0.1-mM dexamethasone, and 20-mM b-glycerophosphate. Matrix mineralization was determined using Alizarin Red S (ARS) (Ricca Chemical) following 21 days of culture in osteogenic medium. Cells were fixed with 10% v/v formalin for 15 minutes, washed once with PBS, and stained with ARS for 20 minutes with gentle rocking. Following this, cells were washed 3 times with PBS and 1 time with molecular grade water, and lastly photographed using a Nikon Eclipse microscope.

Adipogenesis potential was determined after cells were plated as described above and cultured in adipogenic differentiation media for 14 days receiving medium changes every 3-4 days. Adipogenic medium contains standard cell culture medium supplemented with 0.5-mM isobutyl methylxanthine, 50-μM indomethacin, and 0.5-μM dexamethasone. Following 14 days in culture, cells were fixed with 10% v/v formalin for 15 minutes, washed with PBS, and stained with Oil Red O solution (Electron Microscopy Supplies) for 30 minutes with gentle rocking. Cells were washed 3 times with PBS, and photos were taken using a Nikon Eclipse microscope.

For chondrogenesis assay, MSC spheroids were generated by centrifuging 3 × 10^5^ cells at 300× *g* for 5 minutes in 15-mL conical tubes and left in the incubator at 37 °C overnight. The spheroids were subsequently cultured in chondrogenic differentiation medium (MEMalpha supplemented with 1% FBS, 0.1-µM dexamethasone, 0.2-mM ascorbic acid, and 10-ng/mL TGF-β_3_) for 21 days, with a media change every 3 days. The spheroid was then fixed in 10% formalin for 1 hour before staining cartilaginous extracellular matrix using Alcian Blue 8 GX (Millipore Sigma). The stained spheroid was cut in half and loaded onto a glass slide before imaging.

### Wound scratch assay

Phenotypically stabilized human umbilical vein endothelial cells (HUVECs; VeraVecs,^16^ Angiocrine Bioscience) were cultured using the EGM-2 Endothelial Cell Growth Medium-2 BulletKit (Lonza) before seeding for wound/scratch assays. VeraVecs were seeded into 24-well plates with CytoSelect inserts (Cell Biolabs) at 150 000 cells/well (5 replicates per condition) overnight. On the following day, the inserts were lifted and the media was changed to the indicated conditioned media. Images were captured immediately and again after 12 hours. The gap area was quantified using ImageJ, and percent wound healing was calculated using the equation below


$$\begin{array}{l} \frac{\left(Original\;\;\;Gap\;\;\;Area-Final\;\;\;Gap\;\;\;Area\right)}{\left(Original\;\;\;Gap\;\;\;Area\right)}x100. \end{array}$$

### Tube formation assay

HUVECs (VeraVecs) were cultured using the EGM^TM^-2 Endothelial Cell Growth Medium-2 BulletKit. To sensitize the cells, the HUVEC were cultured in DMEM supplemented with 0.2% FBS for 24 hours prior to use. Matrigel Growth Factor Reduced Basement Membrane Matrix (Corning) was added to 24-well plates and allowed to solidify at 37 °C for 1 hour. Sensitized HUVECs were then plated at 50 000 cells/well and allowed to form tubes for 18 hours at 37 °C. For each well, multiple images were taken and stitched together using ImageJ. The combined image was subsequently analyzed using Angiogenesis Analyzer.^17^

### Transcriptome and secretome analysis

To examine the effect of VEGF transgene expression at a transcriptomic level, MSCs (same lot as used for all other studies, but in passage 3) were transduced with either the VEGF lentivirus or with a control lentivirus coding for tdTomato instead of VEGF. These cells were then cultured for either 1 or 2 additional passages. Total RNA was extracted using a commercial kit (Zymo) and submitted to the Gene Expression Core at UC Davis for 3'Tag-based sequencing (Tag-seq). Sequencing results were analyzed by the Bioinformatics core to determine relative expression levels of over 16 000 transcripts, and further downstream analysis, including multidimensional scaling (MDS) plot and unbiased hierarchical clustering.

To determine changes in the secretome of MSCs, we compared untransduced cells to Ruo-MSC/VEGF. Cells were cultured in 225 cm^2^ tissue culture flasks at 2 × 10^6^ cells/flask for 24 hours. Supernatants were collected, treated with Complete Mini—EDTA free protease inhibitor (Roche), and concentrated by centrifugation (30 minutes at 1800× *g*) using 3-kDa cutoff Centricon tubes (Millipore Sigma). Protein concentration was measured using the Bradford method and submitted to the Proteomics Core at UC Davis for proteome analysis using liquid chromatography/mass spectrometry. Bioinformatic analysis was performed by the Core and delivered as relative protein levels.

### Matrigel plugs assay

All animal studies were performed strictly adhering to our approved IACUC protocol. After hypoxic preconditioning, cells were lifted using Trypsin, washed once with PBS, and resuspended in the indicated numbers in Figure 3 in 100 μL of cold Matrigel (to keep it liquid) per mouse. For injections, mice were anesthetized using isoflurane and cell-containing Matrigel was injected subcutaneously into the upper right limb of NSG mice. Sex and age of animals were even across groups. After 14 days, mice were humanely euthanized and Matrigel was carefully removed for photographic documentation.

**Figure 2.**
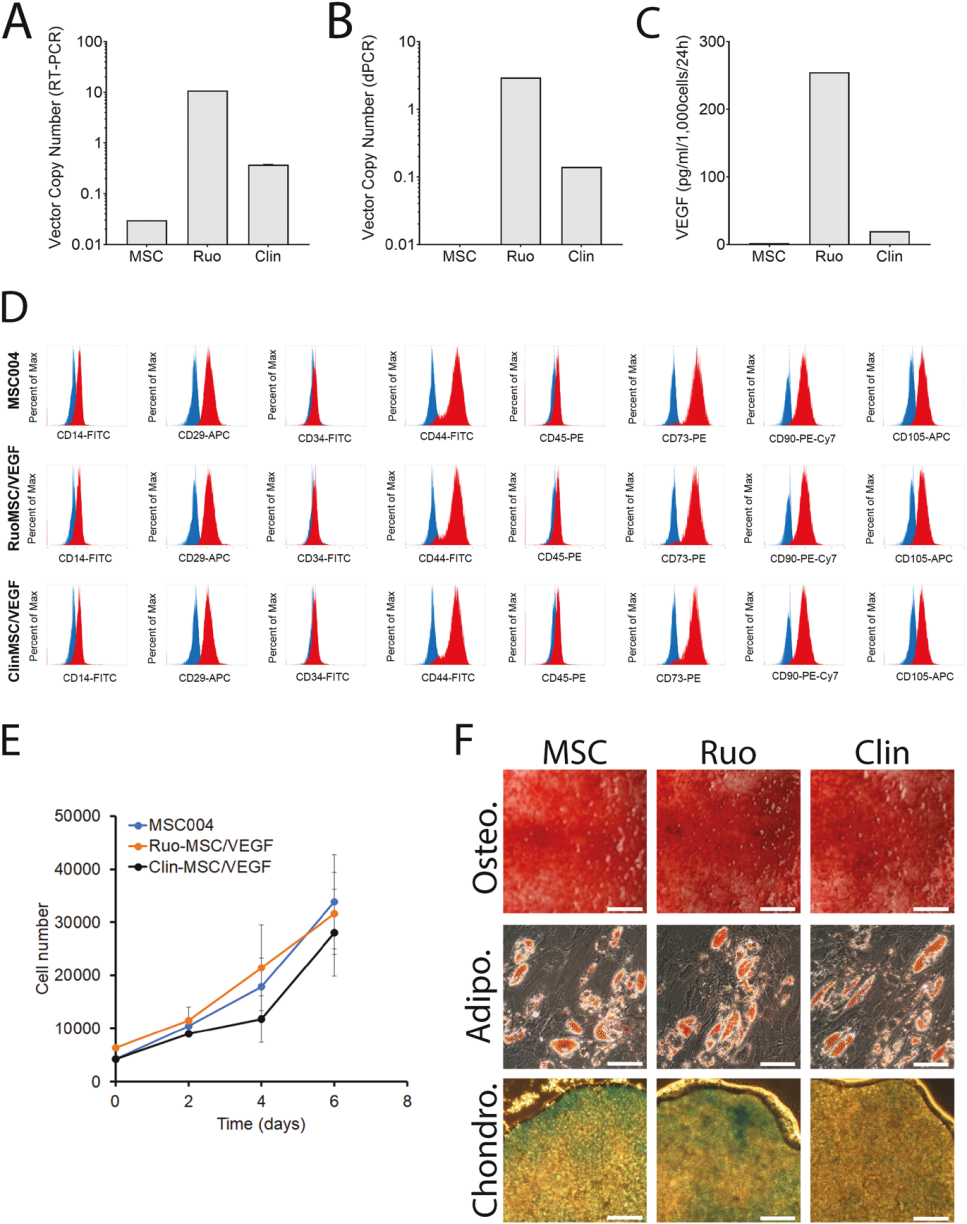
In vitro characterization of MSC/VEGF. (A) Vector copy number (VCN) determined by RT-PCR in unmodified cells (MSC), Ruo-MSC/VEGF (Ruo), and Clin-MSC/VEGF (Clin). (B) VCN determined by dPCR. (C) VEGF secretion measured by ELISA. (D) Immune phenotype measured by flow cytometry. (E) Proliferation measured using hemocytometer and Trypan Blue exclusion dye. (F) Differentiation into osteogenic, adipogenic, and chondrogenic lineages. Bone mineralization was stained using Alizarin Red S, Lipid Droplets in adipocytes are stained with Oil Red O, and cartilage is stained with Alcian Blue. Scale bars for Osteo. are 2 mm, for Adipo. are 100 μm, and for Chondro. are 200 μm.

### Cell retention and distribution

To evaluate retention and distribution of the MSC/VEGF product, 6 immune-deficient NSG mice/sex/time point (84 mice total) received 2 intramuscular injections (quadricep and hamstring) of 5 × 10^5^ of RUO cells per mouse. Following euthanasia at the specified time points, tissue was collected from the quadricep and hamstring muscles of the injected limb, the contralateral limb, and the lungs. Total genomic DNA was extracted using a Quick-DNA Midiprep Plus Kit (Zymo) and real-time PCR was performed using Taqman reagents and the following human ERV3 and mouse GAPDH primers and probes: hERV3-fwd: 5'-CATGGGAAGCAAG-GGAACTAATG-3', hERV3-rev: 5'-CCCAGCGAGCAATACAG AATTT-3', and 5'- 6-fluorescein (FAM)-containing probe 5'-/56-FAM/ TCTTCCCTCGAACCTGCACCATCAAGTCA/36-TAMTSp/-3'. mGAPDH-fwd: 5'-ACCACGAGAAATATGACAA CTCA-3', mGAPDH-rev: 5'-CCCACTGCCTACAT ACCATGAGC-3', and FAM-containing probe 5'-/56-FAM/ TCAGCAATGCATCCTGCACCACCAACT/36-TAMTSp/ - 3'. Mice that were not injected with MSC/VEGF product were used as a negative control (*n* = 3).

### Toxicology studies

For pivotal safety studies, we tested 2 doses, to bracket the intended clinical dose. Based on the area of injection of CLI patients (260 cm^2^) and the area of injection in mice (1.3 cm^2^), the intended high dose for human patients (10^8^ cells) is equivalent to 5 × 10^5^ cells in a mouse. The intended low dose in human patients is 5×10^7^ cells. Therefore, to bracket these doses in mice, we tested 2.5 × 10^5^ cells/mouse (low dose; half of high dose) and 10^6^ cells/mouse (double of high dose). For each of these 2 doses, 10 mice per sex per time point were injected. The total dose (in 100-μL vehicle) was administered over 2 injections per mice, on the back and front of the thigh of the right hind limb. The mice were examined at 3 time points after injection: 2 weeks, 2 months, and 6 months. At each time point, the corresponding group (40 mice total) was sent to the Comparative Pathology Laboratory at UC Davis for complete blood count (CBC), a phenotyping Chemistry Panel, and comprehensive rodent necropsy with histopathology. Tissues for histopathology evaluation were blood, muscle (injection sites), gonads, brain, liver, kidneys, lung, heart, spleen, bone marrow, draining lymph nodes. For the 6 months group, mice’ weight was measured weekly, and animals were monitored daily to closely assess any anomalies.

## Results

### Manufacture of MSC/VEGF

For the manufacturing of MSC/VEGF, MSCs from an allogeneic prequalified suitable bone marrow donor were isolated and expanded in the GMP facility in tissue culture flasks up to passage 2 (P2) and then cryopreserved (Figure 1). The VEGF lentivirus^12^ was also manufactured under GMP and tested for functional titer and sterility. Then, MSCs (P2) were seeded into Terumo Quantum Bioreactors, transduced overnight with the VEGF lentivirus, and further expanded for 6 days for the generation of a Master Cell Bank (MCB) of MSC/VEGF (P3). For the generation of the WCB, cells from the MCB are thawed and plated into 10 bioreactors, expanded for additional 6 days, and cryopreserved. For each expansion in bioreactors, 2 × 10^7^cells would expand to ~2 × 10^8^ cells, a 10-fold expansion comparable to normal expansion in tissue culture flasks.

Various groups have reported increased efficacy using cells that have been cryorecovered prior to infusion, as compared with cells thawed at the bedside.^18-20^ In addition, we and others have shown that hypoxic preconditioning increases cell retention after administration, by altering the metabolic activity of the cells.^21-23^ Therefore, our intended clinical use is that once a suitable patient has been identified and scheduled, cells from the WCB (MSC/VEGF P4) are thawed, plated into CS10 multilayered flasks, cultured for 2 days in hypoxia (2% Oxygen), and finally formulated and loaded into 28 syringes (Figure 1C). The vehicle is saline solution supplemented with 5% HSA.

The GMP facility at UC Davis generated 2 cell banks: A research use only lot (Ruo-MSC/VEGF) that was used for most preclinical studies, and a bank intended for use in CLI patients (Clin-MSC/VEGF). These banks were also compared with each other. The MSCs used in both banks were derived from the same donor (ie, same lot of MSC P2), but a key difference is that Ruo-MSC/VEGF were transduced with higher amounts of VEGF lentivirus (Figure 2), to prioritize modeling a maximum feasible dose, to thoroughly assess safety. Clin-MSC/VEGF were transduced with lower amounts of virus to prioritize clinical safety in patients. The dose-finding studies were performed with Clin-MSC/VEGF, to better predict potential clinical outcomes.

### In vitro characterization of MSC/VEGF

The VCN (average insertions per cell) was measured by RT-PCR and digital PCR (dPCR). RT-PCR of nontransduced MSCs show only background VCN (VCN = 0.03; likely due to unspecific binding), Ruo-MSC/VEGF show an average of 10.8 copies per cell, while Clin-MSC/VEGF (both MCB and WCB) show consistently a VCN of 0.38 (Figure 2A). These results suggest that safety studies were performed with cells that have ~20 times more viral insertions than the clinical lot. Importantly, the number of viral insertions did not change over time, since the MCB (P3) and WCB (P4) show consistently the same VCN (not shown). When measuring VCN using dPCR, the VCN for unmodified MSCs is 0.00, for Ruo-MSC/VEGF is 2.92, and for Clin-MSC/VEGF is 0.14 (Figure 2B). These values differed to what was measured by RT-PCR (using a standard curve) but are consistent in showing that Ruo-MSC/VEGF have on average ~20-fold more viral insertions per cell, as compared with Clin-MSC/VEGF. Importantly, having a VCN below 0.5 for the clinical lot supports the notion of a safe product, due to unlikely genetic alterations caused by the viral insertions.

The amount of VEGF secreted by these cells was also determined. Unmodified MSCs produce around 1.9 pg/mL, while Ruo-MSC/VEGF produces 254 pg/mL. Clin-MSC/VEGF (WCB) produces 19.6 pg/mL (Figure 2C). Therefore, the clinical lot produces 10 times more VEGF than unmodified cells. On the other hand, Ruo-MSC/VEGF produces over 10 times more VEGF than the clinical lot, which is consistent with the higher VCN, and therefore valuable to assess any potential risks caused by excess VEGF.

We next determined if the genetic modification would alter any characteristics of MSCs. In terms of immune profile, the original MSC lot (MSC), Ruo-MSC/VEGF, and Clin-MSC/VEGF (WCB) were virtually identical (Figure 2D), which is the characteristic of MSCs: negative for CD14 (a monocyte marker), CD34 (a marker of hematopoietic stem cells), and CD45 (a pan-hematopoietic marker); and positive for CD29 (integrin b1), CD44, CD73, CD90, and CD105.

We tested if transduction affected proliferation or differentiation of the intended clinical lot. Figure 2E shows that there are no significant differences in proliferation between the lots of cells tested, with an average population doubling time of 40 hours during exponential phase. Similarly, osteogenic, adipogenic, and chondrogenic differentiation were very similar across all 3 lots (Figures 2F). These results are in line with the previous work showing that overexpression of VEGF does not affect the proliferation of differentiation potential of MSCs.^11,12^

To thoroughly exclude an autocrine effect on MSCs due to overexpression of VEGF, we performed both a secretome and transcriptome analysis on MSC/VEGF. Specifically, a transcriptome analysis using Tag-seq and proteome analysis using mass spectrometry were performed to determine how overexpression of VEGF may alter the gene expression profile and secretome of MSCs. For Tag-seq, MSCs were either unmodified, transduced with a control lentivirus, or transduced with the MSC/VEGF lentivirus, each condition under 2 different passages; 4 and 5. An MDS plot and unbiased hierarchical clustering show that overexpression of VEGF had little to no impact on gene expression, as compared with cell passage or manipulation of samples (Supplementary Figure S2A and B).On the proteome analysis, a total of 875 proteins were detected. From these, 158 proteins are secreted proteins, while the remaining proteins were intracellular and likely present in the supernatant due to cell debris or apoptotic cells. Supplementary Figure S2C shows graphically the secretion levels of selected proteins, showing minimal differences in protein levels, with the notable exception of VEGF, which is secreted at supraphysiological levels in Ruo-MSC/VEGF, as compared with unmodified MSCs.

Altogether, our results suggest that overexpression of VEGF had little to no impact on the immune phenotype, proliferation, differentiation, secretome, and transcriptome of MSCs.

### Efficacy and dose-finding studies

**Figure 3.**
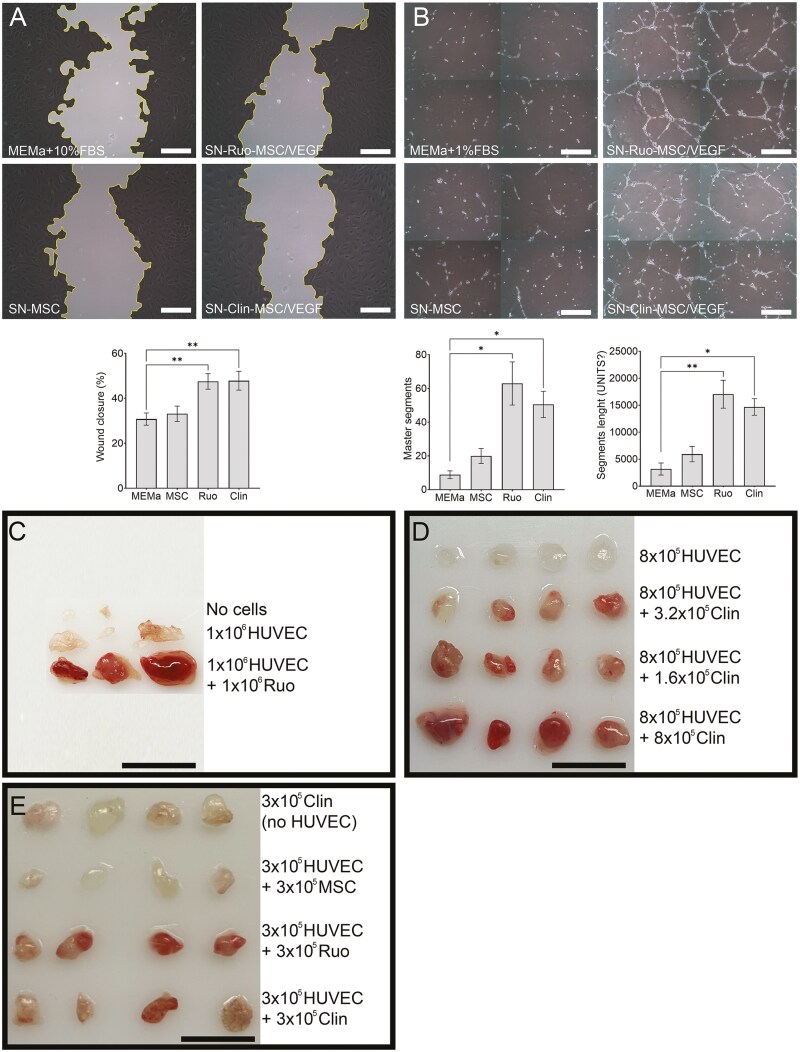
Angiogenic activity of MSC/VEGF. (A) Representative images and quantification of wound/scratch assay using HUVECs cultured for 12 hours with supernatants of unmodified MSCs, Ruo-MSC/VEGF, Clin- MSC/VEGF, or culture media (negative control). Scale bar = 100 μm. (B) Tube formation assays using HUVECs on Matrigel-coated wells and treated with supernatants for 18 hours. Scale bar = 100 μm. (C)–(E) Matrigel plug assays in immune deficient mice. Plugs (100-μl matrigel) were injected into the right hind limb of mice with indicated cells and retrieved for 14 days after. Scale bar in (C)–(E) = 1 cm.

The efficacy of MSC/VEGF in promoting angiogenic activities was first tested in vitro by assessing induction of migration and tube formation of human umbilical cord vein endothelial cells (HUVEC). A wound/scratch assay was used to detect effects on migration of HUVEC. Consistent with previous work,^11,12^ HUVEC showed similar increase in migration when exposed to supernatant from Ruo-MSC/VEGF or Clin-MSC/VEGF, as compared with the negative control and unmodified MSCs (Figure 3A). This demonstrated that overexpression of VEGF in MSCs increases the ability to promote endothelial cell migration. Similarly, tube formation assays suggest that increased angiogenesis in cells exposed to supernatant from Ruo-MSC/VEGF and Clin-MSC/VEGF, as compared with the negative control and the supernatant from unmodified MSC (Figure 3B).

Of note, these studies could not be reproduced using Mouse Aortic Endothelial Cells (MAOEC, iXCells). MAOEC did not form tubes or show increased wound closure in wound/scratch assays, even when treated with mouse endothelial medium (positive control). This observation and previous work showing limited cross-reactivity of human VEGF on murine receptors^24^ prompted the development of an in vivo Matrigel plug assay to test MSC/VEGF in vivo using human endothelial cells.

Based on work published by Shimatani et al.,^25^ an in vivo qualitative angiogenesis assay was developed by injecting subcutaneously Matrigel containing HUVECs and MSC/VEGF. In a first proof-of-concept experiment, we compared 3 experimental groups: (1) Matrigel only (no cells), (2) Matrigel with 10^6^ HUVEC, and (3) Matrigel with 10^6^ HUVEC and 10^6^ Ruo-MSC/VEGF (Figure 3C). Plugs with Matrigel only were very small and clear in color. Plugs with HUVEC were only slightly different. However, the presence of Ruo-MSC/VEGF made the plugs appear much bigger and intensely red, suggesting strong blood perfusion.

Next, we performed a similar in vivo Matrigel plug assay using Clin-MSC/VEGF. Clin-MSC/VEGF were tested in increasing doses with a constant dose HUVEC. As shown in Figure 3D, addition of 3.2 × 10^4^ Clin-MSC/VEGF cells made the Matrigel notably redder, as compared with HUVEC alone, and this effect was increased with higher doses. This study demonstrated that the angiogenic potential of MSC/VEGF is dose dependent.

Finally, we performed an experiment with additional controls to confirm that the effect was driven by signaling of MSC/VEGF to the human endothelial cells, and not to endogenous murine endothelial cells. In addition, we compared nontransduced MSCs to Ruo-MSC/VEGF and Clin-MSC/VEGF, side by side (Figure 3E). Matrigel plugs with Clin-MSC/VEGF but without HUVEC were only minimally pink, suggesting that the injected HUVECs are critical to this ectopic angiogenic plug. The addition of unmodified MSCs had little effect, supporting the notion that unmodified cells are insufficient to promote robust blood formation. Ruo-MSC/VEGF performed the best in terms of generating intensely vascularized Matrigel plugs. Clin-MSC/VEGF performed intermediately since some plugs were very vascularized while others were not. This heterogeneity could be attributed to slight variations in the injection sites; even though all treatments were given by subcutaneous injection in the right hind limb, some plugs may have been placed closer to preexisting vasculature than others. The observation that Ruo-MSC/VEGF promoted more vascularization than Clin-MSC/VEGF is consistent with the higher VEGF secretion levels. This study demonstrates that MSC/VEGF are markedly more angiogenic as compared with unmodified MSCs, supporting that the overexpression of the transgene VEGF is central to the angiogenic effect elicited by the cells.

### Cell retention and biodistribution

To determine the persistence of MSC/VEGF in tissues is a key safety study. Here, animals (*n* = 6/sex/time point) received 2 intramuscular injections of Ruo-MSC/VEGF in the quadriceps and hamstring of the right hind limb. Mice that did not receive cells (*n* = 3; males) served as negative control. For each time point, mice were euthanized, and tissues were collected from muscles of injection site (right hind limb), contralateral muscles of left hind limb, and lungs (a primary site of MSCs lodging if in circulation). We also collected heart and kidney for the 6 months-time point. Total genomic DNA was extracted from the collected tissues to measure the presence of human DNA using RT-PCR. In addition, blood was collected to determine the presence of human VEGF in circulation using a commercial ELISA kit.

**Figure 4.**
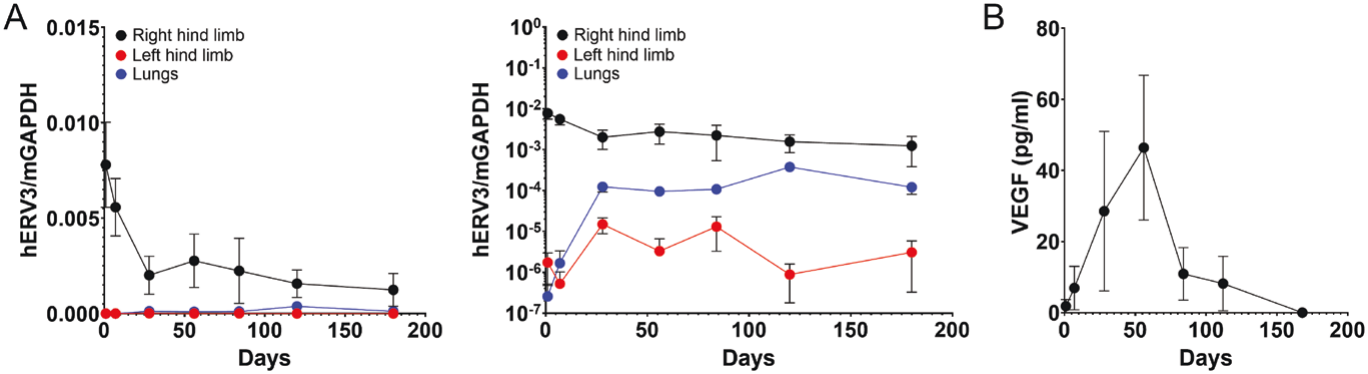
Retention of MSC/VEGF and detection of circulating human VEGF. (A) Presence of Ruo-MSC/VEGF was measured by RT-PCR, by detecting the human-specific ERV3 gene and normalizing to murine DNA (mGAPDH). Each time point is the average of 6 male and 6 female mice, each injected with 5 × 105 cells. The graph on left shows a linear *y-*axis, while the graph in center uses a logarithmic scale. (B) In the same mice used for retention studies also blood was collected to measure circulating VEGF, which was measured by ELISA. A validation of this method is shown in Supplementary Figure S3B.

RT-PCR results, using either a linear scale or logarithmic scale, showed a 4-fold reduction of hERV3 signal within the first 4 weeks (28 days), which then remained rather constant for the remainder of the study. On the noninjected LHL, signal was barely detectable throughout all timepoints. However, the lungs showed an increase in signal during the first 4 weeks, which then also stabilized over time. During this stable phase, the signal in lungs was 10- to 100-fold lower than on the RHL. Six months after cell injection, no significant signal of hERV3 could be detected in either heart or kidneys, suggesting that injected cells remained largely at the injection site (Figure 4A and Supplementary Figure S3). Negative control animals did not show any amplification of hERV3 in any tissue tested (not shown).

Results from the blood analysis showed that the VEGF secreted by MSC/VEGF reached systemic circulation. VEGF levels showed a progressive rise in the bloodstream, reaching their peak at the 8-week mark, followed by a significant decline. After 6 months, VEGF became undetectable in the blood (Figure 4B and Supplementary Figure S4A). The sharp decline in circulating VEGF is correlated with the drop in hERV3 signal detected by RT-PCR, suggesting that the remaining signal of human DNA at later time points is unlikely coming from viable MSC/VEGF.

Altogether, these studies demonstrated that the injected cells primarily remained at the injection site, although a small quantity of cells may have reached circulation to lodge in lungs. Blood analysis revealed that the VEGF secreted by MSC/VEGF reached systemic circulation, peaking at 8 weeks before significantly declining.

### Assessment of potential toxicity

Immunodeficient NSG mice (*n* = 10/sex/dose/timepoint; total 120) were administered 2 intramuscular injections of MSC/VEGF at doses of either 2.5 × 10^5^ cells/mouse (low dose) or 1 × 10^6^ cells/mouse (high dose). Mice were euthanized at 2 weeks, 2 months, or 6 months following injections. Mice were monitored daily and weighed weekly. CBC, a Chemistry Panel, and comprehensive rodent necropsy with histopathology were performed on each animal. For histopathology evaluation, it included blood, muscle (injection sites), gonads, brain, liver, kidneys, lung, heart, spleen, bone marrow, and draining lymph nodes. In addition, we also examined any mice from the retention studies that had signs of potential adverse effects.

At the 2-week time point, all hematological and clinical chemistry parameters for the treatment groups were within the normal reference ranges established by an historical control.

**Figure 5.**
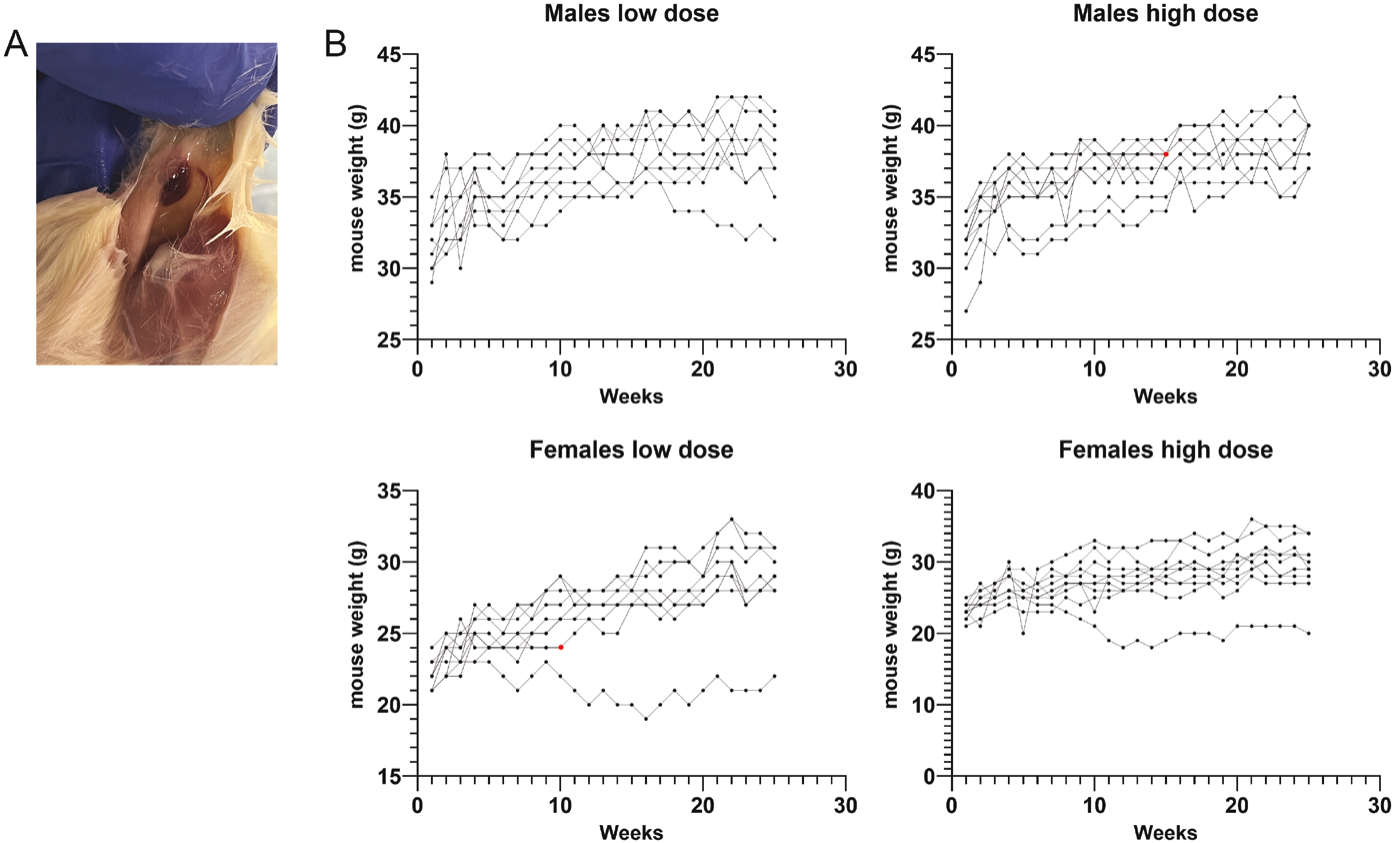
Toxicity studies support safety of MSC/VEGF. (A) Example of a mouse hematoma observed in a subset of mice after 2 months of receiving Ruo-MSC/VEGF. The frequency of these events did not correlate with the dose of cells injected. (B) Mice were checked daily for overall health (not shown) and their weight was measured weekly. The red dots (1 in males receiving high dose [106 Ruo-MSC/VEGF] and 1 in females receiving low dose (2.5 × 105 Ruo-MSC/VEGF) show the last time point before these mice were found dead. However, the cause of death could not be associated to treatment.

At the 2-month necropsy, out of 40 mice, 6 mice (3 receiving low dose and 3 with high dose) showed mild intramuscular bleeding at the injection site. Seven mice (3 receiving low dose and 4 with high dose) showed mild to large intramuscular hematoma at the injection site (Figure 5A). There was no evidence of systemic pathology associated with any treatment group. There were variable amounts of cells within the peri-femoral adipose tissue and peri-femoral skeletal muscle at injection site. These infiltrating cells were variably associated with vague to more discretely formed vascular structures, formation, and severity of which did not appear to correlate with dose. Infiltrating cells (presumably Ruo-MSC/VEGF) were not identified in all evaluated mice.

At 6 months, 2 mice did not complete the study. One female mouse receiving 2.5 × 10^5^ cells was euthanized after 70 days of treatment, because it had lost over 20% of body weight in 1 week prior to euthanasia. The cause of death was undetermined following histological analysis. One male mouse receiving 1 × 10^6^ cells was found dead after 103 days of treatment. The cause of death was not apparent following histological analysis, and there were no significant findings in body weight or clinical signs observed.

For other mice of this cohort, results revealed a regular gain in body weight over the 6-month period (Figure 5B). In hematological parameters, no significant changes were noted in any dose group when compared with the historical control reference ranges. Four mice (2 with low dose and 2 with high dose) showed a hematoma at the injection site and 1 mouse (receiving high dose) showed intramuscular focal bleeding at the injection site. There were no significant organ weight changes specific to a treatment group.

As observed after 2 months, after 6 months, there were also variable amounts of cells identified within the peri-femoral adipose tissue and peri-femoral skeletal muscle that were presumed to be the injected cells. In almost, all animals in which the peri-femoral cells were identified, either suspected or overt vascular formation associated with these cells were noted. Formation of vascular structure did not correlate with dose, though peri-femoral cell infiltrates were most frequently identified in high-dose female mice.

The majority of animals in all groups (17 of 20 from the low-dose group and 17 of 18 from high-dose group) showed minimal to mild cellular infiltrates in the lungs forming small clusters or nodules smaller than 1 mm. Cell types were not always overtly apparent, and in a subset of mice, pulmonary cellular infiltrates were associated with vague to more overt vascular structures. Therefore, consistent with our RT-PCR retention studies, it was suspected that at least some of these infiltrates may be migrating Ruo-MSC/VEGF, though this was not confirmed. Lung cellular infiltrates were scored for severity and distribution, and no trends or significant differences were identified between groups.

Overall, these toxicity studies suggest that MSC/VEGF are well tolerated and show angiogenic properties. There was a regular gain in the body weight of mice over a period of 6 months and no systemic pathology noted at 2 week or 2 months. Two mortalities were noted in the 6-month cohort; however, a correlation with the MSC/VEGF treatment was not established. In fact, similar events also occur sporadically in untreated animals in the UC Davis breeding colony.

## Discussion

A key to a successful IND submission for a novel cell/gene therapy is a fruitful pre-IND interaction, to ensure that the conducted pharmacology and toxicology studies will meet FDA-expected standards. The results presented here are in that line, including the number of animals for each group, time points, and doses tested. The in vitro studies presented here confirm our previous work^11,12^ remaining critical to demonstrate that MSC/VEGF do not differ significantly from regular MSCs, which have extensively shown to be clinically safe.

Although a clear dose-dependent effect on promoting vascularization of Matrigel plugs was seen, it was difficult to infer an optimal clinical dose from these studies, since parameters such as the number of blood vessel occlusions and the size or severity of these occlusions are critical to conducting such an assessment. In fact, because of these uncertainties, we propose to test clinically 2 different doses: 5 × 10^7^ cells and 10 × 10^7^ cells. These numbers are based on previous clinical trials testing up to 6 × 10^8^ MSCs for PAD/CLI.^4,26,27^

The highest level of VEGF detected in circulation was ~200 pg/mL in mice where 5 × 10^5^ cells were injected which would be equivalent to 1.25 × 10^9^ cells in human considering the body weights for mice as 30 g and for human as 75 kg. This calculated dose for humans is over 10 times higher than the dose planned in the proposed clinical trial. Based on these calculations, the maximum VEGF levels that could be detected in patients are still within what are considered normal levels (0-115 pg/mL).^28^ For safe doses of VEGF, Ozawa et al. showed that concentrations over ~100 ng/10^6^ cells/day cause aberrant bulbous structures. Those studies were performed using murine cells overexpressing murine VEGF (A_164_), injected into mice. Therefore, we aimed to manufacture MSC/VEGF that would produce a lower amount than this. Our clinical lot (Clin-MSC/VEGF) produces 45 ng/10^6^ cells/day, while the lot used for all preclinical safety studies (Ruo-MSC/VEGF) produces 555 ng/10^6^ cells/day. Therefore, Ruo-MSC/VEGF (used for safety studies) produce around 10 times more VEGF than the intended clinical lot and the clinical lot produces around 10 times more VEGF than nonmodified MSCs.^12,29^

Another challenge for our studies was the limited cross-reactivity of human VEGF with murine VEGF receptors. Mujagic et al.^24^ showed that human and mouse VEGF react differently to human and mouse endothelial cells. In one in vitro study, they showed that recombinant human VEGF-A (165) is about 4 times less potent than recombinant mouse VEGF (164) on inducing expression of murine VCAM1. This previous work prompted us to test dose-dependent efficacy by co-injecting MSC/VEGF with human endothelial cells, using Matrigel plugs.

It was rather surprising to detect human DNA (hERV3) for most mice at all time points, which is a different outcome to our previously published work^12^ where, using the same method, we detected cells after 4.5 months, but not after 6 months. A potential difference in these assays is that they are semi-quantitative now, but only qualitative previously. The lot of cells and lentivirus used was also different in both cases. It is however clear that retention of MSC/VEGF strongly declines within the first 4 weeks, which is consistent with our previous work using luciferase-based methodology.^12^ Noteworthy, hERV3 signals show an increase in lungs after 4 weeks. Due to limitations of our technique, we do not know if these are living cells or only human DNA. In general, the signal in lungs is 100 times lower than at the injection site.

We speculate that the heterogeneity observed in detection of hERV3 and especially on circulating VEGF levels relates to the exact injection site, as possibly some cells remained closer or more distant to blood vessels. In general, MSCs often generate cell niches with very low cell migration after 10 days.^30^

Based on our toxicology findings, the only potential adverse reaction could be hemorrhages or blood accumulations (likely corresponding to hemangiomas or hematomas) at the injection site. This was observed in 15% of mice after 2 months and in 10% of mice after 6 months and could be attributed to an excess of local VEGF levels. Only one mouse developed a severe adverse event attributable to MSC/VEGF, which was a large hemangiosarcoma with hemorrhage and necrosis. However, it should be noted that the dose tested was very high (with cells producing 10 times more VEGF than the intended clinical lot), that these mice presented normal vasculature (ie, do not present hind limb ischemia), and that the animals are severely immune deficient, making them particularly susceptible to develop tumors.

Finally, the use of immune-deficient mice limits our ability to assess possible effects of the immune system on the modified cells and on the potential effect of MSC/VEGF on fibrosis and inflammation. However, MSCs have a long history of being well tolerated under allogeneic settings,^31^ and the only transgene is human VEGF-A (165), which should not cause immunogenicity. MSCs can exert anti-inflammatory effects^32,33^ that we anticipate will not be affected by the ectopic expression of VEGF.

Altogether, the presented data suggest that MSC/VEGF are more angiogenic than unmodified MSCs and that for the proposed doses tested and target population, these cells will show a good safety profile. Given the urgency to develop new approaches to avoid amputation of limbs of patients with severe CLI, MSC/VEGF is a promising therapeutic to be tested in humans.

## Supplementary Material

szae094_suppl_Supplementary_Figures_S1-S4

## Data Availability

The data underlying this article will be shared on reasonable request to the corresponding author.
