# Supplementary material for: Investigational New Drug-enabling studies to use genetically modified mesenchymal stromal cells in patients with critical limb ischemia
Source: Stem Cells Transl Med. 2025 Feb 26;14(2):szae094. doi: 10.1093/stcltm/szae094 (PMC11878639; doi:10.1093/stcltm/szae094)
Supplement: szae094_suppl_Supplementary_Figures_S1-S4 [file szae094_suppl_supplementary_figures_s1-s4.docx]

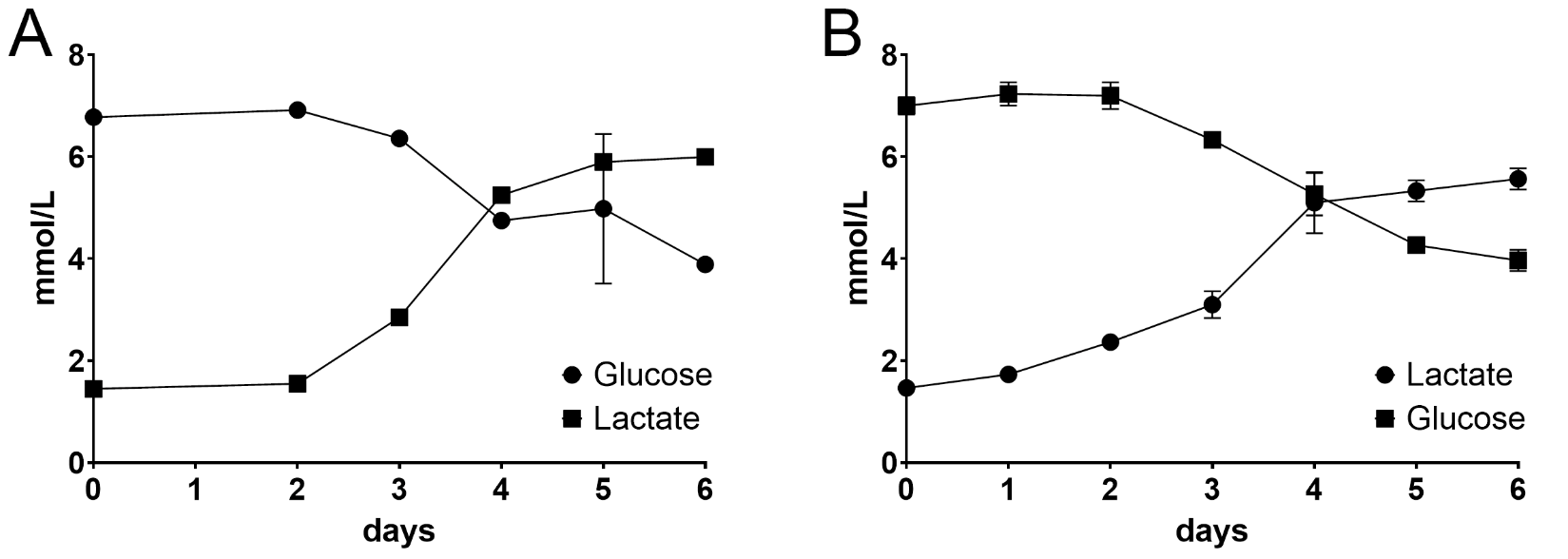


**Supplementary Figure S1. Glucose and lactate measurements of Clin/MSC/VEGF in Terumo Quantum Bioreactors.** Glucose and lactate were monitored daily during each expansion. (A) MCB (n = 2). (B) WCB (n = 10). In both cases, the plateau in lactate levels reached at day 6, suggested the end of the exponential expansion phase, prompting the harvest of cells for cryopreservation.


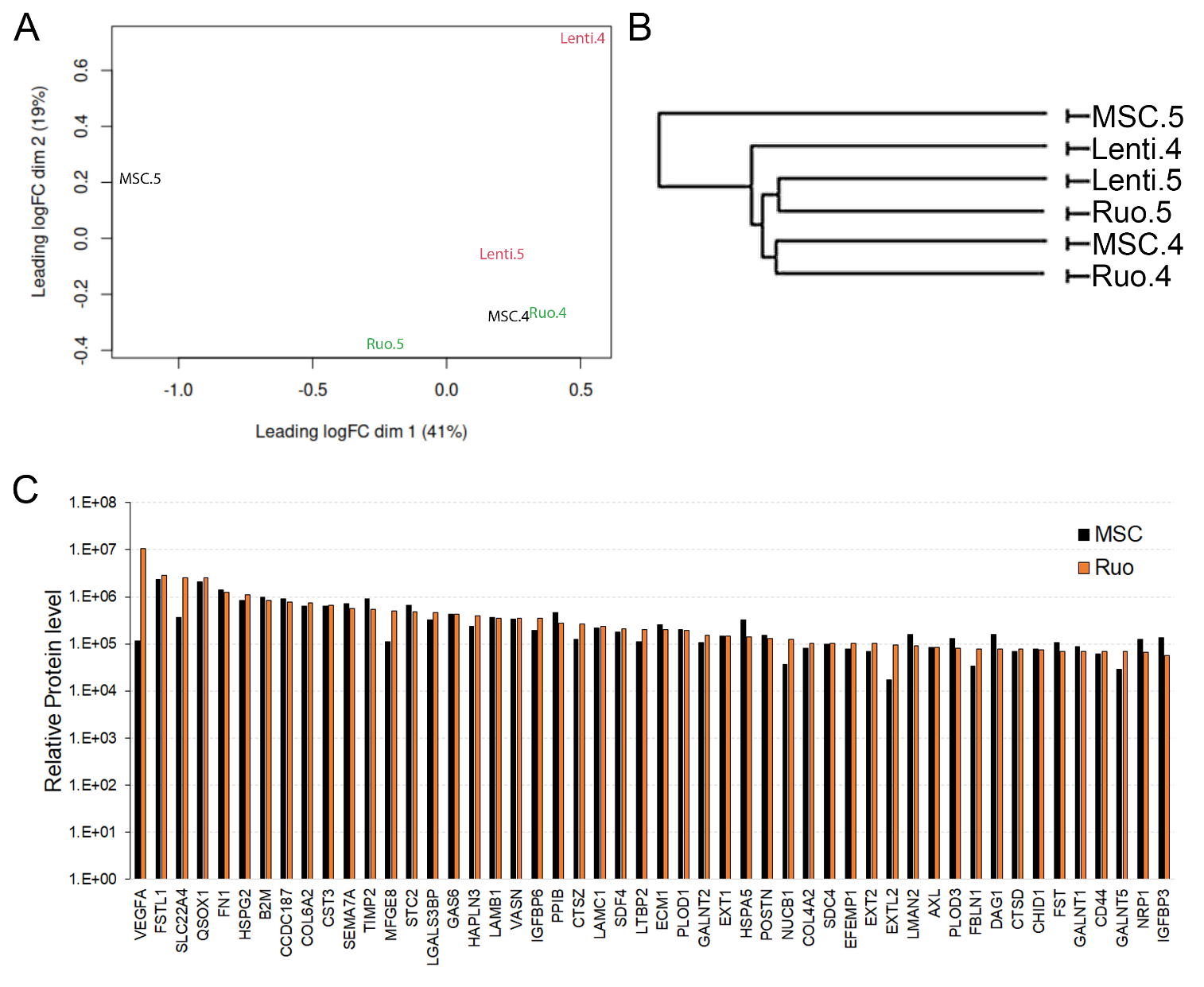


**Supplementary Figure S2. Transcriptome and secretome comparison of MSCs and Ruo-MSC/VEGF.** (A) MDS plot of gene expression profiles measured using Tagseq, in MSCs either unmodified (MSC), Ruo-MSC/VEGF (Ruo), or transduced with a control lentivirus (Lenti), each at either Passage 4 or 5. (B) Hierarchical clustering of samples. The largest differences in gene expression were observed in samples MSC.5 and Lenti.4, suggesting that over-expression of VEGF has a minimal impact on global gene expression in MSCs. (C) Top 50 most abundant proteins secreted by MSC vs. MSC/VEGF, as measured using mass spectrometry. Notice the strong increase in VEGFA levels, while other detected proteins show rather modest differences.

**
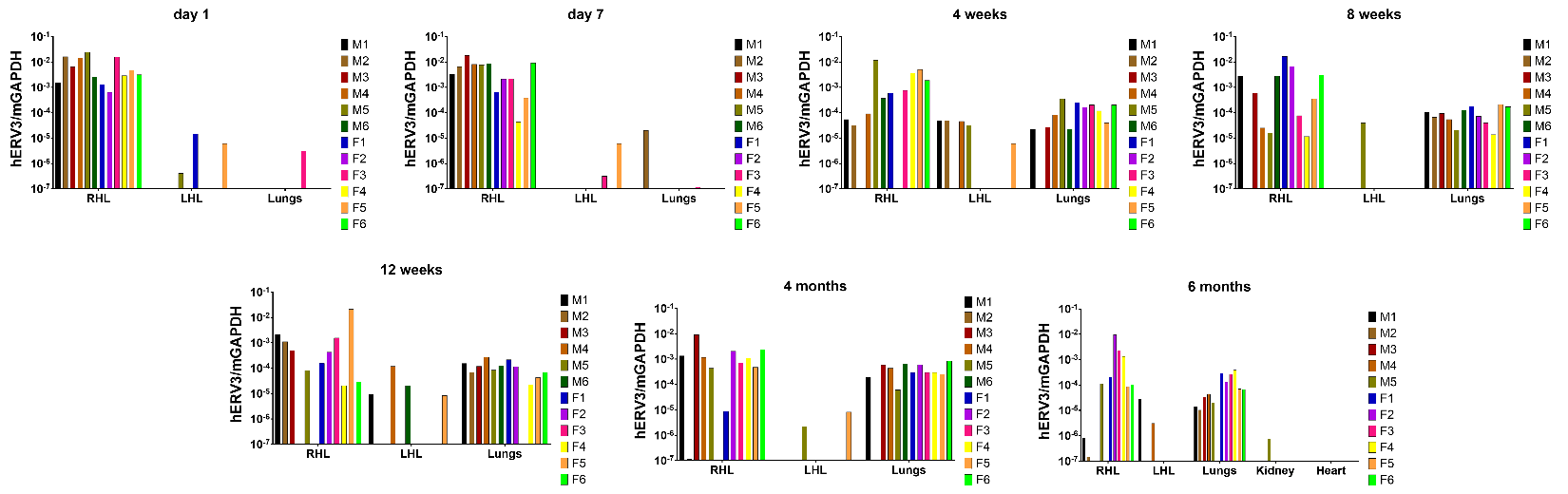
**

**Supplementary Figure S3. Detection of Ruo-MSC/VEGF in mice.** Related to Figure 4A, here we show the detection of human DNA (as a surrogate to measure persistence of Ruo-MSC/VEGF) in individual mice. M=male, F=female, RHL=right hind limb (injection site), LHL=left hind limb.

**
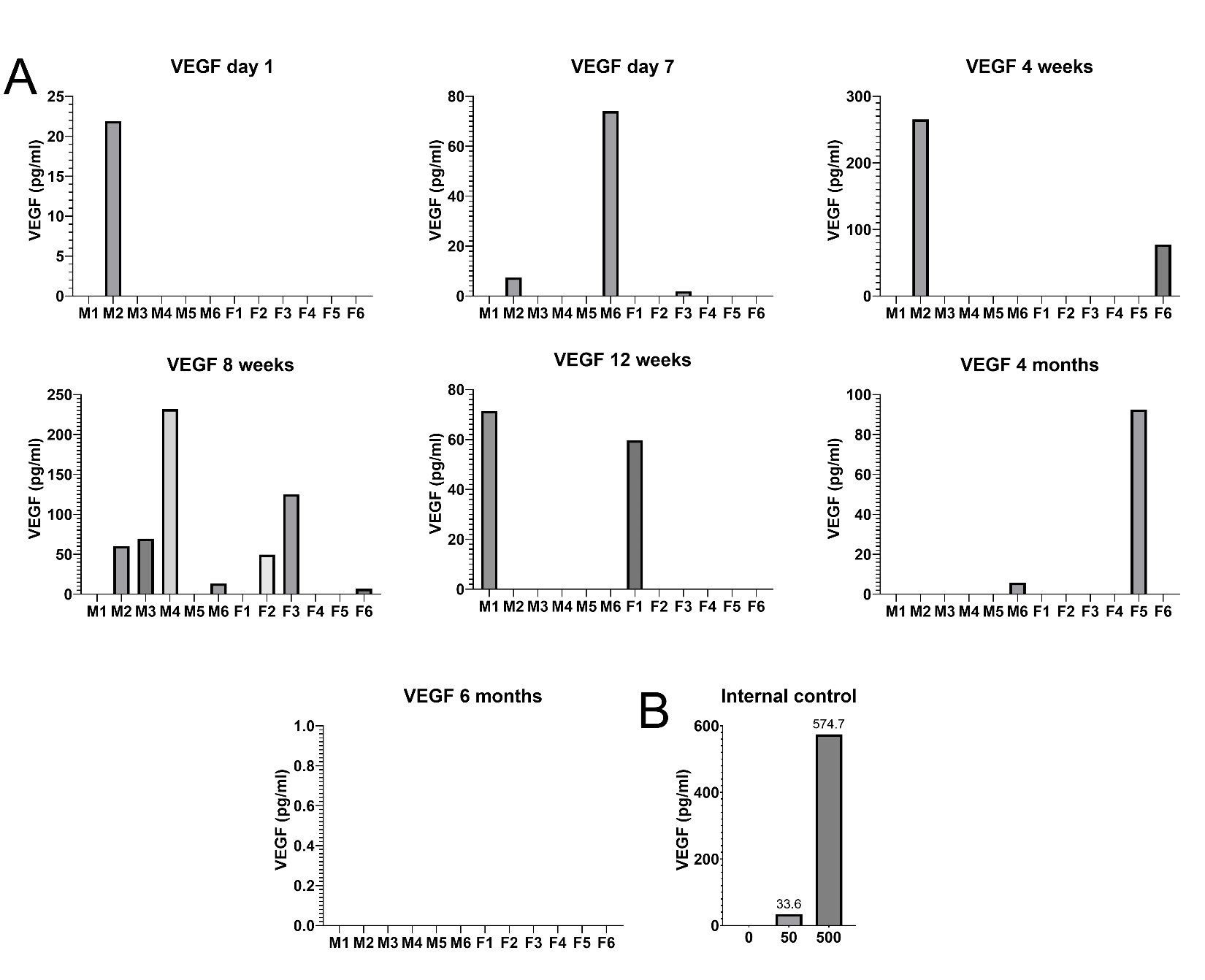
**

**Supplementary Figure S4. Detection of VEGF in circulation.** (A) Related to Figure 4B, this figure shows detection of VEGF in individual mice. (B0 Internal control (validation study) showing that the detection of VEGF closely aligns with the quantity added (pg/ml in X axis). VEGF levels were not detected in negative control (murine blood without addition of VEGF), or in murine blood spiked with 10 pg/mL of human VEGF, highlighting the limitations in sensitivity of this ELISA kit.
